# Weight Loss and Percutaneous Endoscopic Gastrostomy Tube Placement during Chemoradiotherapy for Locally Advanced Cancer of the Oropharynx Do Not Negatively Impact Outcomes

**DOI:** 10.3389/fonc.2017.00299

**Published:** 2017-12-05

**Authors:** Michael J. Baine, Timothy Dorius, Nathan Bennion, Lynette Smith, Weining Zhen, Apar Kishor Ganti

**Affiliations:** ^1^Department of Radiation Oncology, Fred and Pamela Buffett Cancer Center, University of Nebraska Medical Center, Omaha, NE, United States; ^2^Department of Medical Oncology, Fred and Pamela Buffett Cancer Center, University of Nebraska Medical Center, Omaha, NE, United States; ^3^Center for Collaboration on Research Design and Analysis, Department of Biostatistics, University of Nebraska Medical Center, Omaha, NE, United States

**Keywords:** head and neck cancer, squamous cell carcinoma, oropharyngeal cancer, toxicity, chemoradiotherapy

## Abstract

**Objectives:**

Concurrent chemoradiotherapy is standard of care in locally advanced oropharyngeal cancer (LA-OPC). This treatment regimen results in significant acute toxicities. This study investigates the effect of treatment-related toxicity on patient outcomes.

**Methods:**

Patient information was retrospectively collected for patients treated for LA-OPC between 2007 and 2014. Factors analyzed included age, gender, pretreatment ECOG performance status, smoking history, patient BMI prior to and following treatment, tumor histology, disease stage, disease recurrence, incidence, and timing of feeding tube placement, radiation dose received, chemotherapy regimen used and if it was completed, and patient survival. All statistical analysis was provided through the University of Nebraska Medical Center Department of Biostatistics.

**Results:**

74 patients were identified with a median follow-up of 3.4 years and a median age of 58.5. Most patients were male (87.8%) and had squamous cell histology (98.7%). Most patients underwent chemoradiotherapy alone (98.6%) and received concurrent cisplatin (78.4%) with approximately half (53.4%) receiving all planned chemotherapy. Upon multivariate analysis, both disease-free (DFS) and overall survival (OS) rates were improved by lower pretreatment BMI, increased weight lost during treatment, and lack of percutaneous endoscopic gastrostomy (PEG) tube placement prior to treatment initiation. Neither DFS nor OS was impacted by placement of a PEG tube during active treatment.

**Conclusion:**

These data suggest that weight loss and PEG tube placement during chemoradiotherapy for LA-OPC, presumably due to treatment-associated mucositis and xerostomia, are not associated with worse outcomes.

## Introduction

Oropharyngeal cancer (OPC) comprises approximately 1% of all cancers diagnosed in the USA with an estimated 15,520 new cases diagnosed in 2015 resulting in 2,660 deaths. This malignancy is predominately diagnosed in males with a male to female incidence of 2:1 (1). This overall incidence has remained relatively steady over time, though associated patient demographics have shifted significantly over the past 30 years.

While OPC is classically of squamous cell histology, its etiology is considered dichotomous with causative factors directly contributing to patient outcomes. Specifically, the nature of OPC is accepted to be directly related to the presence of absence of HPV. HPV non-related OPC is often attributable to tobacco and alcohol exposure and is associated with worse overall survival (OS) despite having a proportionally lower risk of nodal and distant metastases (2). HPV 16 and 18 are the most common strains linked with malignancy and are thought to be acquired through sexual contact (3). The prevalence of HPV-associated OPC has been steadily increasing since the mid-1970s with tobacco-related OPC remaining steady until the mid-1980s followed by a significant decline thereafter (4).

Treatment of OPC is determined by stage, with division into three groups usually considered: early stage, locally advanced, and distantly metastatic. By definition, early stage OPC consists of clinical stages I or II based on the American Joint Committee on Cancer (AJCC) Staging Manual, 7th edition while locally advanced consists of stage III–IVB disease (5). Per National Comprehensive Cancer Network guidelines, recommended options for the initial treatment of early stage OPC include definitive radiation therapy (RT) or resection. Surgery alone can be appropriate for early stage disease or patients with early stage primary tumors and limited associated pathologic lymphadenopathy. In locally advanced OPC (LA-OPC), recommended initial treatment options include definitive RT combined with systemic agents, resection followed by either radiation or chemoradiotherapy depending on pathologic features, or induction chemotherapy followed by either definitive radiation or chemoradiotherapy (3, 6). In distant metastatic disease, as is the case in most malignancies, chemotherapy remains the backbone of treatment, occasionally with the addition of palliative treatment to the primary tumor or other sites of symptomatic disease.

For patients with early stage OPC, both resection and definitive RT are often tolerated relatively well with toxicities that, while at times severe, are usually manageable. In LA-OPC, however, the combination of systemic agents and RT results in a significant increase in both short-term and long-term side effects. The concurrent systemic therapy utilized with radiation for LA-OPC most often consists of three doses of cisplatin 100 mg/m^2^ though can alternatively consist of weekly cisplatin, carboplatin or, more recently, cetuximab (7–13). While these agents themselves have associated toxicities, combination with RT can result in increased mucositis which can result in poor oral intake, increased weight loss, increased treatment breaks due to associated pain, dehydration, and/or electrolyte abnormalities, and placement of a percutaneous endoscopic gastrostomy (PEG) tube (14, 15). Additionally, concurrent chemotherapy and radiation increases the risk of long-term treatment-associated toxicities such as pharyngeal dysfunction and long-term requirement of PEG tube placement (16).

The effect that these toxicities have on treatment outcomes remains unclear. Indeed, even theoretical postulation in this regard can be difficult as clear arguments can be made from both a positive and negative standpoint. This is particularly true with regard to acute toxicities such as mucositis and its associated odynophagia and resultant weight loss; more severe toxicity may represent a more robust treatment response in both the tumor and surrounding tissues, though the associated pain and anorexia prevents many patients from completing their prescribed chemotherapy regimen and results in breaks from treatment during RT, potentially compromising the treatment itself (17–22).

## Materials and Methods

### Patients

Data were collected from patient treatment databases and corresponding Electronic Medical Records for patients who underwent combined chemotherapy and RT for LA-OPC at the University of Nebraska Medical Center (UNMC). Inclusion criteria included the patient being diagnosed with stage III–IVB OPC, receiving treatment between the years 2007 and 2014, and receiving both chemotherapy and RT through UNMC. Disease-specific factors that were analyzed included tumor histology as well as disease stage and incidence of disease recurrence following completion of therapy. p16 status was not available for a significant number of patients (19 patients, 26.0%) in our dataset and those with unknown HPV status were analyzed as a separate patient group. Other patient factors that were collected and analyzed included age, gender, ECOG performance status prior to treatment initiation, smoking history, pretreatment BMI, posttreatment BMI, incidence and timing of feeding tube placement, and death. Regarding BMI, both raw BMI number and classification were utilized in our analysis. With regard to PEG tube placement, out institutional policy is to encourage PEG tubes for all patients who have lost ≥10% of their pre-diagnosis body weight though, in our experience, many patients are reluctant to undergo this procedure and thus percent weight lost prior to placement may be higher than 10% in some individuals. All patients at our institution are referred to Speech Pathology for aid in maintenance of swallowing function both prior to and following PEG placement. Patients are additionally encouraged to continue following with Speech Pathology after treatment completion with ultimate discharge from the service per the discretion of the treating therapist. Treatment-specific factors included radiation dose received, chemotherapy regimen used, and number of cycles of chemotherapy provided. Data collection and project design were approved by the UNMC Institutional Review Board.

### Statistics

All statistical analysis was provided through the UNMC Department of Biostatistics (Lynette Smith). OS and disease-free survival (DFS) were estimated *via* the Kaplan–Meier method. The log-rank test was used to compare survival distributions between groups. OS was defined as years from diagnosis to death or last follow-up. DFS was defined as years from diagnosis to recurrence, death, or last follow-up. Data for age, radiation dose, pretreatment BMI, posttreatment BMI, and percent BMI lost were considered continuous variables. Patient characteristics such as patient gender, ECOG performance status, smoking history, histology, BMI classification before and after treatment, changing of BMI classification over the treatment course, placement of PEG tube prior to treatment initiation, placement of PEG tube during treatment, tumor stage, nodal stage, whether the patient underwent surgical resection, completion of the prescribed RT course, completion of all prescribed cycles of chemotherapy, type of chemotherapy received, local recurrence, regional recurrence, distant recurrence, and death were all considered categorical variables. Multivariate analysis was performed using Cox regression. *p*-Values of <0.05 were considered statistically significant.

## Results

### Patient Population

In total, 73 patients were found to meet the inclusion criteria of this study. Median follow-up was 3.4 years with follow-up ranging from 0.6 to 8.7 years. The median age of diagnosis was 58.7 years and 87.7% of the patients analyzed were males (Table 1). Of the 67 patients whose pretreatment performance status was recorded, 65.7% had an ECOG performance status of 0 and 32.8% had a performance status of 1 while a single patient had a recorded performance status of 2. More than half of the analyzed patients (57.8%) were current or former smokers. All but one patient was diagnosed with squamous cell carcinoma with the singular outlier being diagnosed with undifferentiated carcinoma. The majority of patients had tumor stages of T2–T4 with nodal stages of N2a–N2b per AJCC 7th edition staging criteria (Table 1).

**Table 1:** Characteristics of patients, disease, treatment, and outcomes (*n* = 73).

Characteristic	Variable	Frequency	Percent
**Patient and disease characteristics**			
Age (years)	Mean (SD)	58.7	(9.3)
Gender	Male	64	87.7
HPV status	Positive	46	63.0
	Negative	8	11.0
	Not documented	19	26.0
BMI category at start of treatment (kg/m^2^)	18.5–24.99	15	20.8
	25–29.99	23	31.9
	≥30	34	47.2
BMI category at treatment completion (kg/m^2^)	<18.5	1	1.4
	18.5–24.99	29	40.3
	25–29.99	28	38.9
	≥30	14	19.4
Change in BMI category (start to completion)	No change in category	38	52.8
	Dropped 1 category	32	44.4
	Dropped 2 categories	2	2.8
BMI at start of treatment (kg/m^2^)	Mean (SD)	29.9	(5.7)
BMI at treatment completion (kg/m^2^)	Mean (SD)	26.6	(4.9)
BMI lost during treatment	Mean (%)	3.23	(10.8)
Pretreatment ECOG	0	44	65.7
	1	22	32.8
	2	1	1.5
	Unknown	6	
Smoking status	Never smoker	30	42.3
	Current/former smoker	41	57.8
Histology	Squamous cell carcinoma	72	98.6
	Undifferentiated	1	1.4
T-stage	*in situ*	1	1.4
	1	5	6.9
	2	34	46.6
	3	17	23.3
	4	15	20.5
	X	1	1.4
N-stage	0	2	2.7
	1	3	4.1
	2	66	90.4
	3	2	2.7

**Treatment characteristics**			
Completed radiation treatment	Yes	71	97.3
Radiation dose (cGy)	Median (range)	7,000	(5,200–7,116)
Completed chemotherapy	Yes	39	54.2
Chemotherapy regimen	Every 21-day cisplatin	57	78.1
	Cetuxumab	15	20.6
	Other	1	1.4

**Treatment morbidity and outcome**			
PEG tube placement	Before chemo/RT	11	15.1
	During chemo/RT	31	42.5
	Never	31	42.5
Recurrence	Local only	1	1.4
	Local + regional	1	1.4
	Regional only	2	2.7
	Regional + distant	1	1.4
	Distant only	8	11.0
	No	60	82.2
Died	Yes	18	24.7
	No	51	69.9
	Unsure	4	5.5

The median radiation dose provided was 7,000 cGy with all patients receiving at least 5,200 cGy. All but two analyzed patients completed their prescribed radiation course. The majority of patients (78.1%) were treated with an every-21-day cisplatin regimen with another 20.6% being treated with weekly cetuximab instead. Just over half of the patients (54.2%) received all planned chemotherapy during treatment (Table 1).

At the start of treatment, most patients were noted to be overweight (31.9%) or obese (47.2%) with no patient being underweight. Over the course of treatment, patient BMIs decreased by a mean of −3.23 kg/m^2^ (−10.8%), resulting in 44.4% of patients dropping by one BMI category and 2.8% of patients dropping two categories (Table 1). No patient was found to move up in BMI category over the course of treatment. PEG tubes were placed in 42 patients with 11 requiring placement prior to initiation of chemoradiotherapy. Two patients (2.8%) experienced a local recurrence, four (5.5%) experienced a regional recurrence, and nine (12.4%) experienced a distant recurrence following therapy. In total, 18 patients (24.7%) had died at the end of follow-up with another 4 (5.5%) having an unknown living status (Table 1).

### Factors Affecting DFS

**Figure 1:**
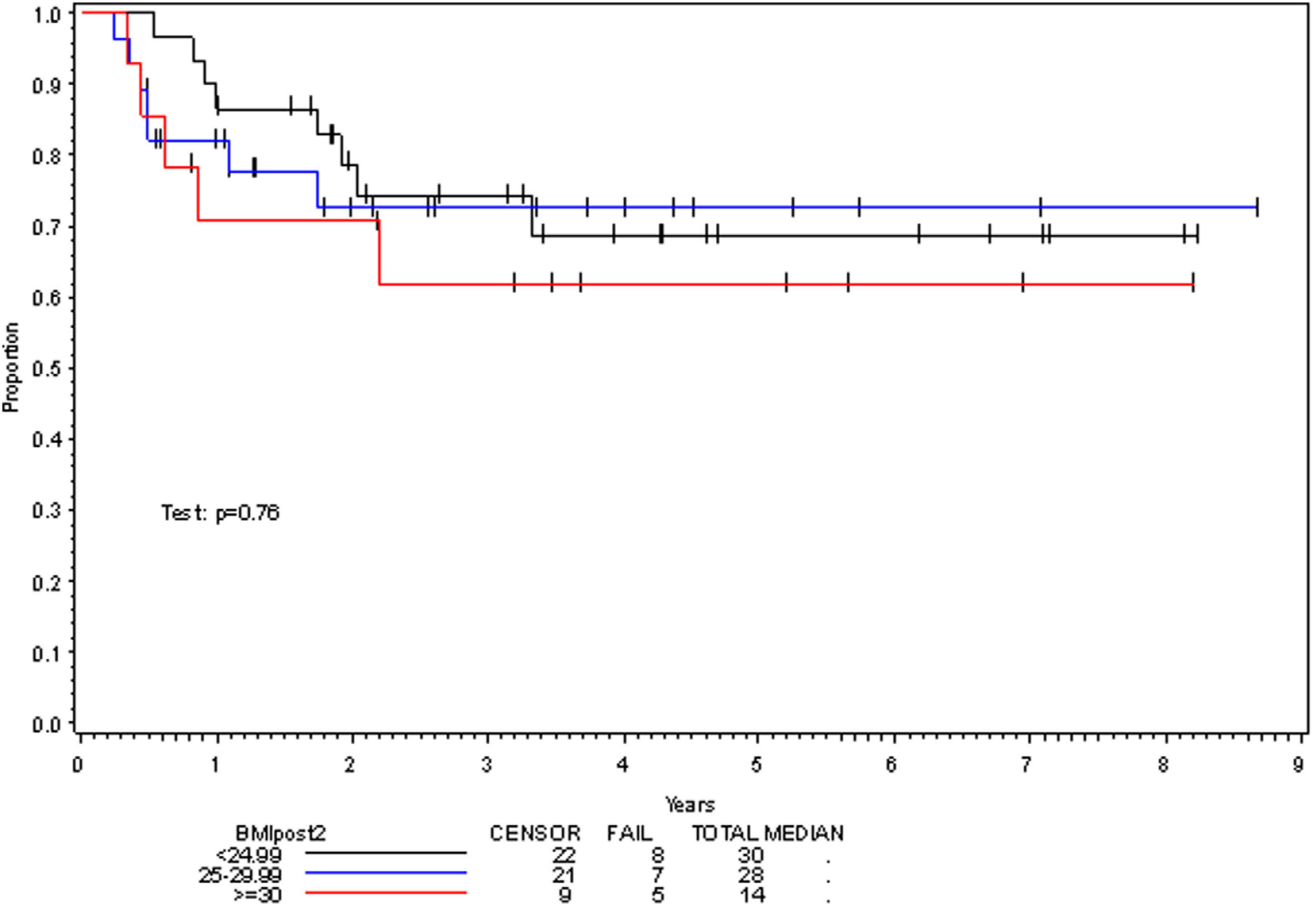
Disease-free survival per posttreatment BMI category.

**Figure 2:**
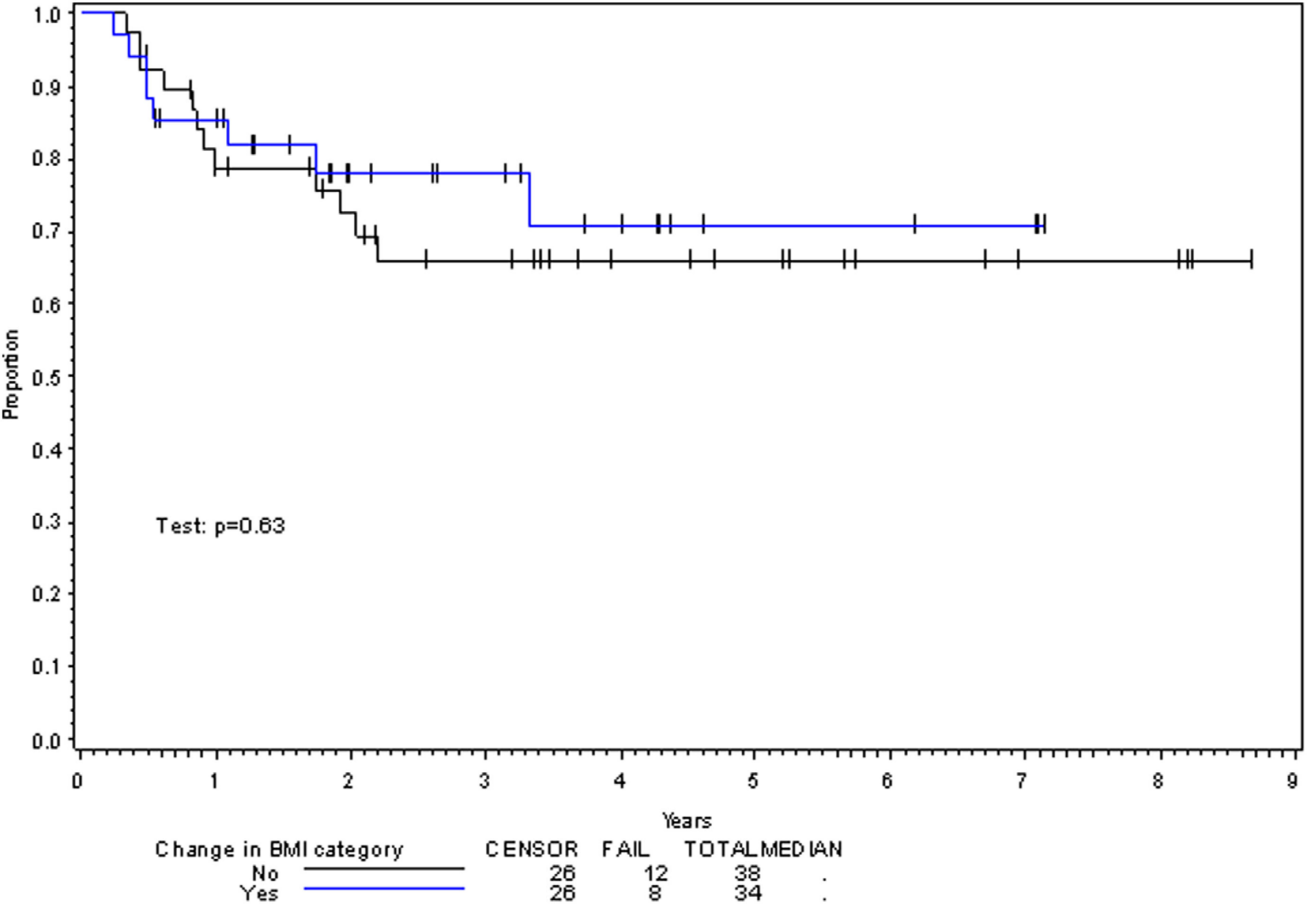
Disease-free survival per change in BMI category with treatment.

On multivariate analysis, factors associated with increased toxicity were found to confer either a significant improvement in, or have no effect on, DFS. Specifically, increased relative BMI reduction improved DFS with each percent decrease in BMI found to result in a hazard ratio (HR) for disease recurrence of 0.85 (95% CI 0.73–0.99, *p* = 0.045) (Table 2). Posttreatment BMI category and movement across categories were not associated with DFS (Figures 1 and 2). Additionally, placement of a PEG during chemoradiotherapy did not appear to affect DFS (*p* = 0.30). Interestingly, however, PEG placement prior to initiation of therapy was associated with an increased risk of disease recurrence with an HR of 9.85 (95% CI 1.59–60.8, *p* = 0.014). Conversely, increased BMI at the start of therapy was also found to be associated with reduced DFS with an HR of 1.15 for each kilogram per square meter increase in BMI (95% CI 1.00–1.33, *p* = 0.047) (Table 2). Patient age at diagnosis, gender, pretreatment performance status, past history of smoking, and HPV status were not associated with DFS in the analyzed patient population (Table 2). Of note, patient T-stage, chemotherapy type (cisplatin vs. cetuximab), and completion of all prescribed chemotherapy were not associated with altered DFS (data not shown) in any multivariate modeling tested in this patient set. These data were not included in the presented multivariate analysis in an attempt to limit the number of included variables to preserve analytical integrity.

**Table 2:** Multivariate analysis of association with time to disease recurrence.

Category	Variable		95% confidence interval		
		HR	Lower	Upper	*p*-Value
Age at diagnosis	1-year increase	1.06	0.98	1.14	0.17
Gender	Male (vs. female)	34.2	0.81	1.438	0.064
ECOG	1 or 2 (vs. 0)	0.77	0.21	2.79	0.69
Smoking status	Current or former smoker (vs. non-smoker)	1.68	0.53	5.35	0.38
HPV p16	Negative (vs. positive)	1.44	0.28	7.29	0.66
	Not documented (vs. positive)	1.94	0.49	7.72	0.35
	Negative (vs. not documented)	0.74	0.11	5.10	0.76
BMI at RT start	1-U increase	1.15	1.00	1.33	0.049
Percent BMI loss	1% greater loss	0.85	0.73	1.00	0.045
PEG timing	Placed prior to treatment initiation (vs. no)	9.85	1.58	60.6	0.014
	Placed during treatment (vs. no)	0.43	0.09	2.11	0.30

*HR, hazard ratio; RT, radiation therapy; PEG, percutaneous endoscopic gastrostomy tube*.

### Factors Affecting OS

**Figure 3:**
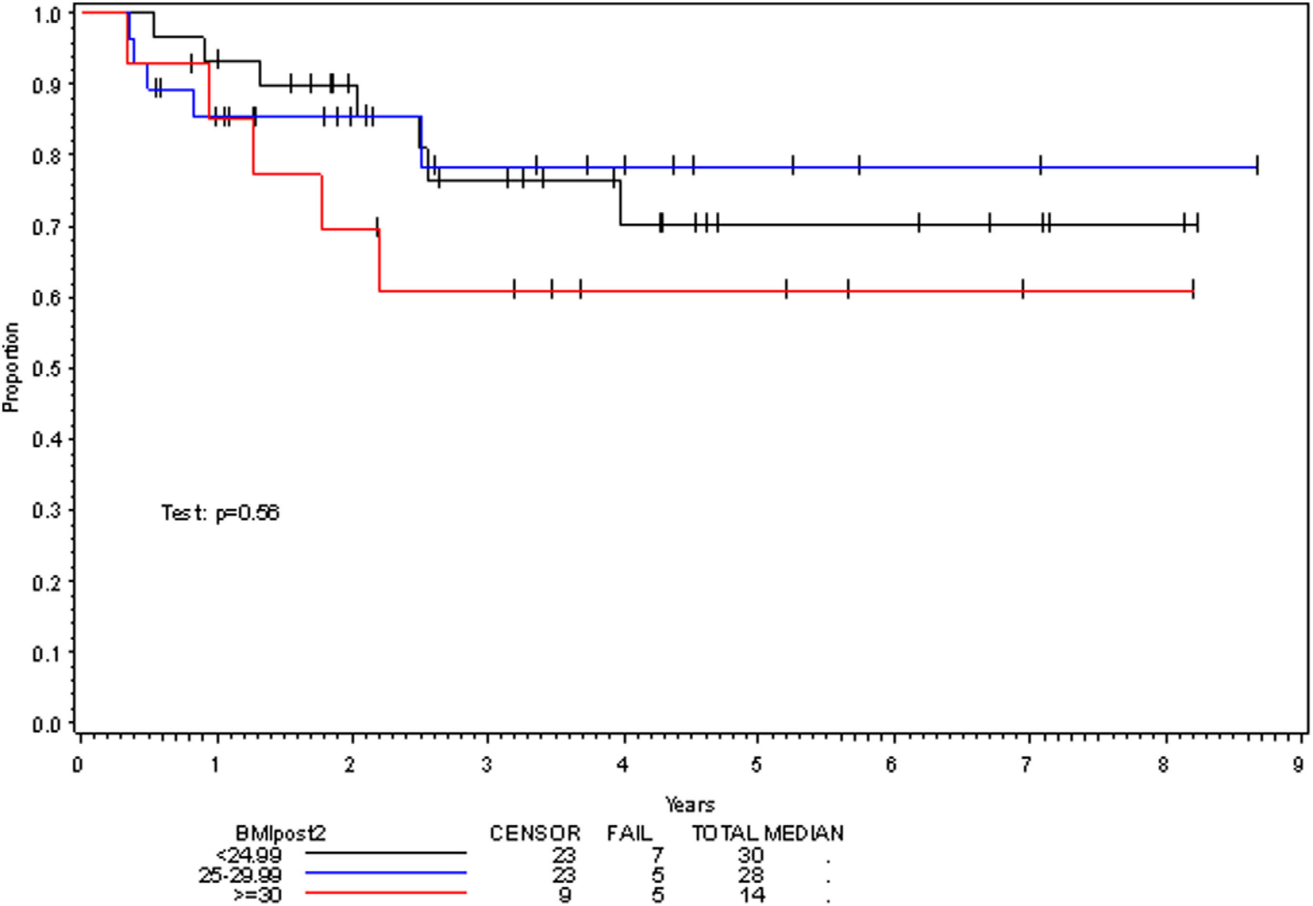
Overall survival per posttreatment BMI category.

**Figure 4:**
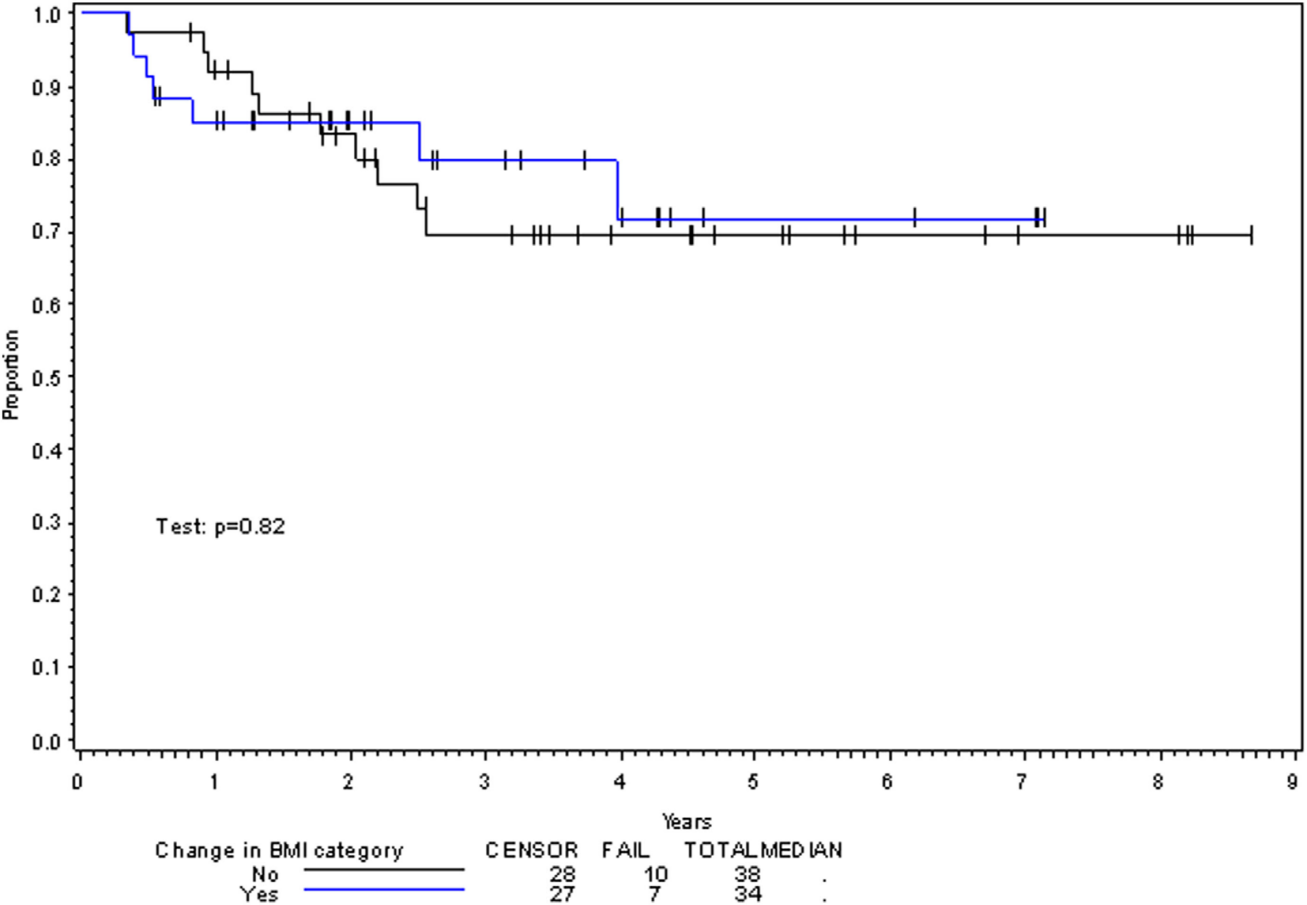
Overall survival per change in BMI category with treatment.

Multivariate analysis for OS was similar as well. Reduction in BMI during chemoradiotherapy was significantly associated with improved survival (HR of death 0.78; 95% CI 0.66–0.93, *p* = 0.005). Interestingly, higher BMI at the time of initiation of treatment was associated with decreased survival with an HR of death of 1.20 (95% CI 1.04–1.38, *p* = 0.012) (Table 3). However, neither BMI category at the time of treatment completion nor change in BMI category during treatment appeared to affect OS (Figures 3 and 4). PEG placement during chemoradiotherapy was not associated with a change in OS though placement prior to treatment initiation was associated with an OS detriment with an HR of death of 11.62 (95% CI 1.77–76.63, *p* = 0.011).

**Table 3:** Multivariate analysis of association with time to death.

Category	Variable		95% confidence interval		
		HR	Lower	Upper	*p*-Value
Age at diagnosis	1-year increase	1.09	1.00	1.18	0.044
Gender	Male (vs. female)	13.21	0.55	315.6	0.11
ECOG	1 or 2 (vs. 0)	0.98	0.26	3.66	0.97
Smoking status	Current or former smoker (vs. non-smoker)	0.86	0.26	2.87	0.81
HPV p16	Negative (vs. positive)	1.77	0.28	11.28	0.55
	Not documented (vs. positive)	3.36	0.84	13.43	0.087
	Negative (vs. not documented)	0.53	0.07	4.02	0.54
BMI at RT start	1-U increase	1.20	1.04	1.38	0.012
Percent BMI loss	1% greater loss	0.78	0.66	0.93	0.0054
PEG timing	Placed prior to treatment initiation (vs. no)	11.62	1.77	76.47	0.011
	Placed during treatment (vs. no)	0.43	0.08	2.24	0.32

*HR, hazard ratio; RT, radiation therapy; PEG, percutaneous endoscopic gastrostomy tube*.

An additional factor associated with improved OS included younger age at diagnosis with an HR of death being 1.09 (95% CI 1.00–1.18, *p* = 0.044) per year of increased age. Interestingly, male gender, pretreatment ECOG performance status, smoking history, and HPV status were not significantly associated with OS, though there was a trend toward improved survival in HPV-positive patients when compared with those with unknown HPV status (*p* = 0.087) (Table 3). Similar to DFS, T-stage, chemotherapy type, and completion of all prescribed chemotherapy were also not associated with OS and thus not included in the presented multivariate analysis (data not shown).

## Discussion

The results of this study provide insight regarding the interplay between toxicity and treatment outcome in LA-OPC. Interestingly, this research suggests that weight loss and PEG tube placement, presumably due to mucositis and xerostomia associated with treatment with chemoradiotherapy, are not associated with worse outcomes. Conversely, relative BMI reduction during treatment correlated with improved DFS as well as OS in LA-OPC patients receiving chemoradiotherapy. Additionally, patients with a lower BMI at the start of chemoradiotherapy had improved DFS and OS in our patient population. Furthermore, while PEG tube placement during therapy did not alter the analyzed patient outcomes, placement of a PEG tube prior to treatment initiation resulted in a substantial increase in both disease recurrence and death. This finding is in direct contrast with the fact that pretreatment ECOG performance status did not alter either DFS or OS, a result which may be due to there being only a single patient with a recorded performance status >1. The association of younger age with improved DFS and OS is consistent with that seen in other data sets and provides some indication of the validity of the presented data (23). Interestingly, while tobacco history and HPV status are well documented to be associated with decreased DFS and OSs, these factors did not affect outcomes in our patient population (24). The result is likely attributable to the fact that quantified past tobacco exposure in pack-years was not widely available for patients included in our dataset, thus allowing for confounding variables to remain unaccounted for as well as a large proportion of the included patient population being without known HPV status. Upon analysis of our data, it does appear that the patients with undocumented HPV status had similar DFS and OS characteristics to those known to be HPV-negative, likely indicating that HPV was absent in the majority of these patients.

Potential explanations for the results of this study are varied. The direct association between relative BMI reduction and both DFS and OS may signal a more robust treatment response in patients with greater treatment-associated toxicity that is present not just in the healthy tissues but in malignant tissues as well. This raises the possibility that patients with greater toxicity from chemoradiotherapy for LA-OPC have radiosensitizing biologic factors which remain present in their respective cancers as well. The association of pretreatment PEG tube placement and increased risk of both disease recurrence and death appears to not be representative of general worse health in these patients as outcomes were not found to be associated with pretreatment performance status. Rather, this finding may be due to association with more advanced stage, problematic tumor locations, increased comorbidities, and greater likelihood for treatment breaks. Lastly, explanation of the relationship between lower pretreatment BMI and improved DFS and OSs may signal that high-BMI states are associated with high-risk characteristics not corrected for in this study such as poor immune response or altered tumor oxygenation patterns. Alternatively, the association of lower BMI at the start of treatment with improved OS in LA-OPC patients may be representative of increased comorbidities associated with increased BMI as opposed to a direct link with cancer-specific treatment outcomes as BMI can act as a surrogate for overall health. Of further consideration is that the influence of BMI may be different between those who are HPV-positive and those who are not. Higher pretreatment BMI in the HPV-negative group may be indicative of a lower disease burden and thus reduced tumor-associated weight loss whereas it may be associated with poorer overall health and a greater number of associated comorbidities in those who are HPV-positive.

This study has several caveats. First and foremost, we recognize the bias inherent in retrospective studies. Lack of information regarding patient comorbidities or actual cause of death may confound the analysis in this study. Further, lack of detailed information on the cause of PEG tube placement prior to treatment initiation prevents detailed analysis to further explain the observed association of pretreatment PEG placement and patient outcomes. Additionally, the included patient population was too small to allow for more detailed analysis of specific OPC subsites and how BMI reduction and PEG tube placement may differentially affect patient outcomes across these. The small sample analyzed and low number of events decrease the robustness of the associated multivariate analyses and make extrapolation of the findings of this study to larger patient populations difficult. Additionally, lack of information on HPV status prevents its analysis with regards to treatment outcomes in this patient set, precluding interpretation of how well this data can be extrapolated to other populations as well.

While the presented data are limited, it provides fodder for multiple avenues of further study which may have significant clinical implications. The association of increased weight loss with improved outcomes seen in this analysis merits further evaluation with larger retrospective and prospective studies to better assess the universality these results. Examination into the biological and biochemical aspects of patient tumors and healthy tissues as they are associated with both treatment-related toxicity and clinical outcome is also warranted. Such research may provide further insight into both the mechanism underlying the observations is this study as well as provide potential future targets for radiosensitization of LA-OPC and/or radioprotection of the surrounding tissues. Additionally, further analysis into the effect of patient comorbidities and cause of death would allow for improved interpretation of the association of higher pretreatment BMI with reduced OS seen in the current study and may also allow for improved counseling of patients with respect to prognosis.

## Ethics Statement

All data collection and project design were approved by the Institutional Review Board at the University of Nebraska Medical Center. Following IRB review, it was determined that patient consent was not required for this study.

## Author Contributions

MB, TD, NB, WZ, and AG conceptualized the study. MB, TD, and NB performed all data collection. LS performed all data analysis. MB wrote the manuscript and organized/produced all tables and figures. TD, NB, LS, WZ, and AG edited the manuscript.

## Conflict of Interest Statement

The authors declare that the research was conducted in the absence of any commercial or financial relationships that could be construed as a potential conflict of interest.

## References

[1] American Cancer Society. Cancer Facts & Figures 2017. Atlanta: American Cancer Society (2017).

[2] Nguyen-Tan PF Zhang Q Ang KK Weber RS Rosenthal DI Soulieres D Randomized phase III trial to test accelerated versus standard fractionation in combination with concurrent cisplatin for head and neck carcinomas in the Radiation Therapy Oncology Group 0129 trial: long-term report of efficacy and toxicity. J Clin Oncol (2014) 32(34):3858–66. 10.1200/JCO.2014.55.3925 25366680 PMC4239304

[3] Ang KK Garden AS Radiotherapy for Head and Neck Cancers: Indications and Techniques. 4th ed Philadelphia: Lippencott Williams & Wilkins (2012).

[4] Chatervedi AK Engels EA Anderson WF GIllison ML Incidence trends for human papillomavirus-related and -unrelated oral squamous cell carcinomas in the United States. J Clin Oncol (2008) 26(4):612–9. 10.1200/JCO.2007.14.1713 18235120

[5] Edge SB Byrd DR Compton CC Fritx AG Green FL Trotti A, editors. AJCC Cancer Staging Manual. 7th ed France: Springer (2010).

[6] Head and Neck Cancers. NCCN Clinical Practice Guidelines in Oncology. (2016). Available from: http://www.nccn.org, Version I

[7] Pignon JP Bourhis J Domenge C Designe L Chemotherapy added to locoregional treatment for head and neck squamous cell carcinoma: three meta-analyses of updated individual data. Lancet (2000) 355:949–55. 10.1016/S0140-6736(00)90011-4 10768432

[8] Wendt TG Grabenbauer GG Rödel CM Thiel HJ Aydin H Rohloff R Simultaneous radiochemotherapy versus radiotherapy alone in advanced head and neck cancer: a randomized multicenter study. J Clin Oncol (1998) 16:1318–24. 10.1200/JCO.1998.16.4.1318 9552032

[9] Calais G Alfonsi M Bardet E Sire C Germain T Bergerot P Randomized trial of radiation therapy versus concomitant chemotherapy and radiation therapy for advanced-stage oropharynx carcinoma. J Natl Cancer Inst (1999) 91:2081–6. 10.1093/jnci/91.24.2081 10601378

[10] Adelstein DA Lavertu P Saxton JP Secic M Wood BG Wanamaker JR Mature results of a phase III randomized trial comparing concurrent chemoradiotherapy with radiation therapy alone in patients with stage III and IV squamous cell carcinoma of the head and neck. Cancer (2000) 88:876–83. 10679658 10.1002/(sici)1097-0142(20000215)88:4<876::aid-cncr19>3.0.co;2-y

[11] Olmi P Crispino S Fallai C Torri V Rossi F Bolner A Locoregionally advanced carcinoma of the oropharynx: conventional radiotherapy vs. accelerated hyperfractionated radiotherapy vs. concomitant radiotherapy and chemotherapy—a multicenter randomized trial. Int J Radiat Oncol Biol Phys (2003) 55:78–92. 10.1016/S0360-3016(02)03792-6 12504039

[12] Budach V Stuschke M Budach W Baumann M Geismar D Grabenbauer G Hyperfractionated accelerated chemoradiation with concurrent fluorouracil-mitomycin is more effective than dose-escalated hyperfractionated accelerated radiation therapy alone in locally advanced head and neck cancer: final results of the radiotherapy cooperative clinical trials group of the German Cancer Society 95-06 prospective randomized trial. J Clin Oncol (2005) 23:1125–35. 10.1200/JCO.2005.07.010 15718308

[13] Bensadoun RJ Bénézery K Dassonville O Magné N Poissonnet G Ramaïoli A French multicenter phase III randomized study testing concurrent twice-a-day radiotherapy and cisplatin/5-fluorouracil chemotherapy (BiRCF) in unresectable pharyngeal carcinoma: results at 2 years (FNCLCC-GORTEC). Int J Radiat Oncol Biol Phys (2006) 64:983–94. 10.1016/j.ijrobp.2005.09.041 16376489

[14] Vera-Llonch M Oster G Hagiwar M Sonis S Oral mucositis in patients undergoing radiation treatment for head and neck carcinoma: risk factors and clinical consequences. Cancer (2006) 106(2):329–36. 10.1002/cncr.21622 16342066

[15] Adelstein DJ Oropharyngeal cancer: the role of chemotherapy. Curr Treat Options Oncol (2003) 4(1):3–13. 10.1007/s11864-003-0027-6 12525275

[16] Machtay M Moughan J Trotti A Garden AS Weber RS Cooper JS Factors associated with severe late toxicity after concurrent chemoradiation for locally advanced head and neck cancer: an RTOG analysis. J Clin Oncol (2008) 26(21):3582–9. 10.1200/JCO.2007.14.8841 18559875 PMC4911537

[17] Dorius T Bennion N Smith L Van Briggle B Zhen W Ganti A Effect of weight loss during concurrent chemoradiation on outcomes of oropharyngeal cancer. J Clin Oncol (2016) 34(Suppl):abst reply 17541 10.1200/JCO.2016.34.15_suppl.e17541

[18] Baine MJ Dorius T Bennion N Alam M Smith L Zhen W Chemoradiotherapy for locally advanced squamous cell carcinoma of the oropharynx: does completion of systemic therapy affect outcomes? Oral Oncol (2017) 73:105–10. 10.1016/j.oraloncology.2017.08.015 28939061

[19] Fayette J Molin Y Lavergne E Montbarbon X Racadot S Poupart M Radiotherapy potentiation with weekly cisplatin compared to standard every 3 week cisplatin chemotherapy for locoregionally advanced head and neck squamous cell carcinoma. Drug Des Devel Ther (2015) 9:6203–10. 10.2147/DDDT.S81488 PMC4664534 26648696

[20] Bahig H Fortin B Alizadeh M Lambert L Filion E Guertin L Predictive factors of survival and treatment tolerance in older patients treated with chemotherapy and radiotherapy for locally advanced head and neck cancer. Oral Oncol (2015) 51:521–8. 10.1016/j.oraloncology.2015.02.097 25797461

[21] Krstevska V Stojkovski I Zafirova-Ivanovska B. Concurrent radiochemotherapy in locally-regionally advanced oropharyngeal squamous cell carcinoma: analysis of treatment results and prognostic factors. Radiat Oncol (2012) 7:78. 10.1186/1748-717X-7-78 22640662 PMC3404949

[22] Adelstein DJ Adams GL Wagner H Kish JA Ensley JF Schuller DE An intergroup phase III comparison of standard radiation therapy and two schedules of concurrent chemoradiotherapy in patients with unresectable squamous cell head and neck cancer. J Clin Oncol (2003) 21:92–8. 10.1200/JCO.2003.01.008 12506176

[23] SEER Fast Stats. (2016). Available from: http://Seer.cancer.gov

[24] Mallen-St. Clair J Alani M Wang MB Srivastan ES Human papillomavirus in oropharyngeal cancer: the changing face of a disease. Biochim Biophys Acta (2016) 1866:141–50. 10.1016/j.bbcan.2016.07.005 27487173

